# Diagnostic and therapeutic approach to obscure gastrointestinal bleeding in a patient with a jejunal gastrointestinal stromal tumor: a case report

**DOI:** 10.1186/1756-0500-7-695

**Published:** 2014-10-07

**Authors:** Jonathan B Yuval, Gideon Almogy, Victoria Doviner, Miklosh Bala

**Affiliations:** Department of General Surgery, Hadassah-Hebrew University Medical Center, POB 12000, Jerusalem, 91120 Israel; Department of Pathology, Hadassah-Hebrew University Medical Center, Jerusalem, Israel

**Keywords:** GIST, Gastro-intestinal, Bleeding, Obscure

## Abstract

**Background:**

Gastrointestinal stromal tumors of the alimentary tract may present with severe bleeding. Localization and treatment of obscure gastrointestinal bleeding is challenging in cases of negative bi-directional endoscopy.

**Case presentation:**

A previously healthy 64-year-old Caucasian female presented with clinical signs of active gastrointestinal bleeding. Esophagogastroduodenoscopy was normal, and colonoscopy revealed passage of blood from the small bowel. Computerized tomography angiography demonstrated a hypervascular lesion with active extravasation located in the jejunum. Angiography of the superior mesenteric artery revealed a focal hypervascular mass in the jejunum, and super selective distal coil embolization of the feeding vessel was performed. When the patient was taken for laparoscopic exploration, a 2.5 cm tumor arising from the anti-mesenteric border of the proximal jejunum was identified and resected with primary anastomosis. Pathological results demonstrated a gastrointestinal stromal tumor with a low proliferation index of 1%. Small erosions in the adjacent mucosa confirmed the locus of bleeding.

**Conclusions:**

Computerized tomography is a useful tool for initial diagnosis of submucosal alimentary tumors in patients with obscure but clinically overt gastrointestinal bleeding. Selective angiography, following positive computerized tomography findings, is an important modality to allow both localization and hemostasis in actively bleeding small bowel tumors, but the procedure carries the risk of bowel necrosis. Complete surgical resection remains the mainstay for treatment of gastrointestinal stromal tumors.

## Background

Gastrointestinal stromal tumors (GISTs) are rare mesenchymal neoplasms of the gastrointestinal (GI) tract. They account for 0.3 to 0.5% of all GI tumors [1] and may be found anywhere along the GI tract, but predominantly develop in the stomach and small intestine [2]. GISTs usually present with vague abdominal symptoms; severe overt bleeding is a rare presentation of this disease [2]. Although there are adjuvant medical therapies, only complete surgical resection is curative [3].

In this paper we report a case of obscure, overt GI bleeding due to a jejunal GIST. We discuss our pre-operative diagnostic steps and management and provide a brief review of the literature on GIST and obscure GI bleeding.

## Case presentation

**Figure 1:**
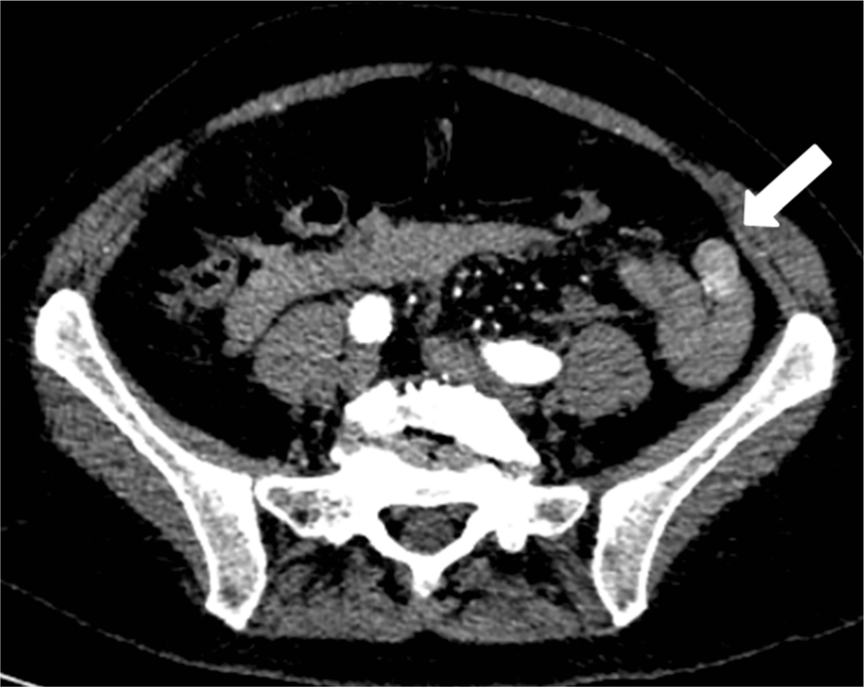
**Computerized tomography angiography demonstrated a hypervacularized small bowel tumor.** The arrow is pointing to the tumor. Upon close observation contrast extravasation into the lumen can be seen.

**Figure 2:**
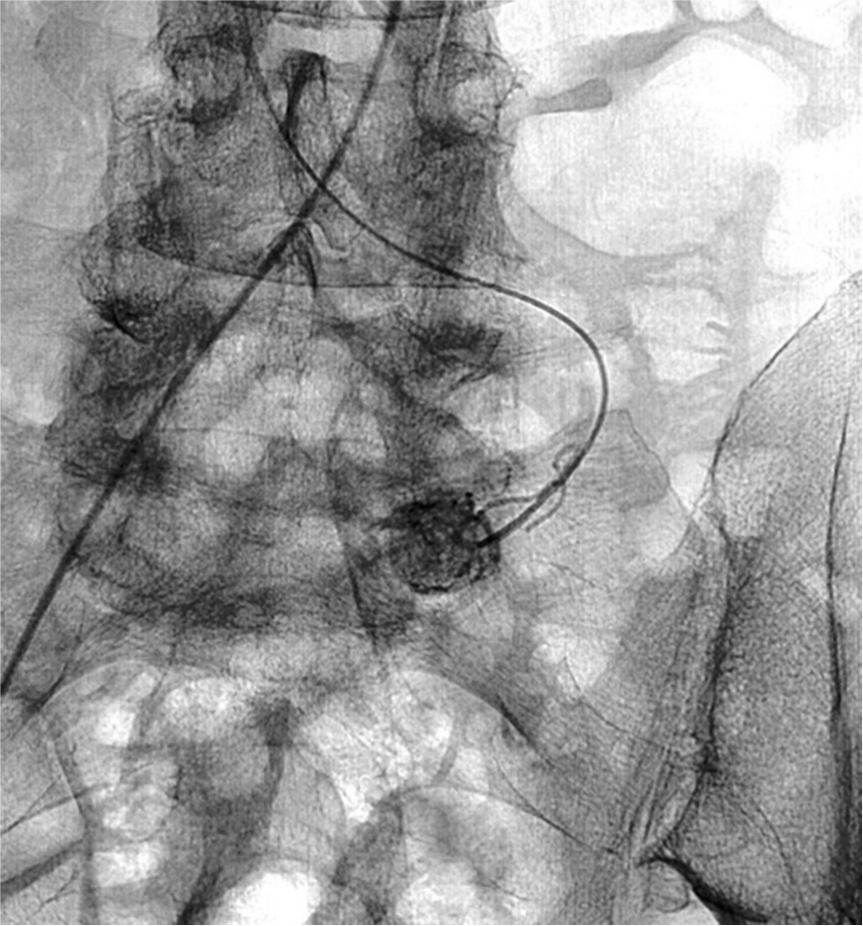
**Diagnostic angiography revealed a hypervascularized jejunal mass.**

**Figure 3:**
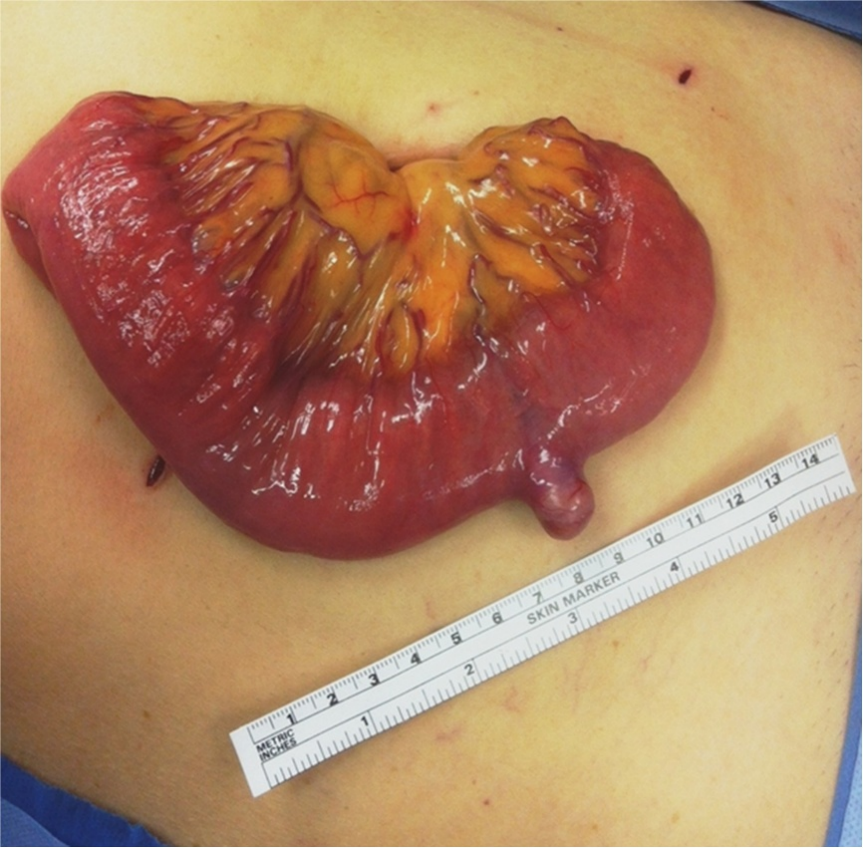
**Antimesenteric jejunal tumor found during surgery.**

**Figure 4:**
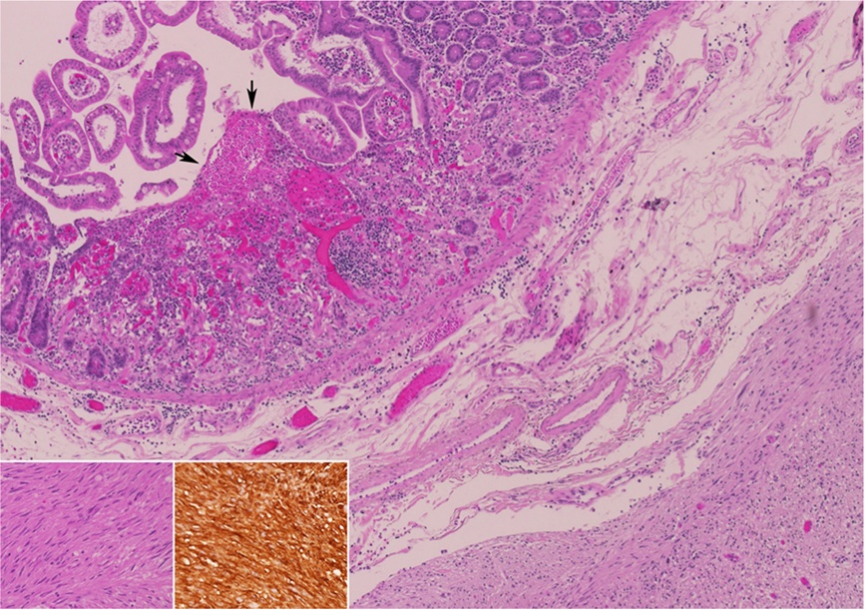
**Pathology of the tumor was consistent with gastrointestinal stromal tumor.** Small intestine with an intramural gastrointestinal stromal tumor, causing (arrows) ischemic erosion of the overlying mucosa (Hematoxylin and Eosin, original magnification X40). The tumor is composed of (inset a) fascicles of spindle cells with bland nuclei, presenting (inset b) immunoreactivity for CD117 (a - Hematoxylin and Eosin, original magnification X200, b – CD117, original magnification X200).

A previously healthy 64-year-old Caucasian female presented to the emergency department (ED) with melena and syncope. She showed signs of hypovolemia with a heart rate of 110 beats per minute and a blood pressure of 100/50 mmHg. Digital rectal examination demonstrated hematochezia. A nasogastric tube was inserted and revealed clear gastric contents. Initial hemoglobin and hematocrit were 9 gr% and 26% respectively. Platelet count was normal. Subsequent blood counts demonstrated a drop in hemoglobin and hematocrit to 7 gr% and 20%, accompanied by a mild thrombocytopenia of 100*10^9^/L. She received two units of packed red blood cells and was started on intravenous proton pump inhibitors. Esophagogastroduodenoscopy (EGD) was normal up to the third part of the duodenum, while colonoscopy demonstrated passage of blood from the terminal ileum without identifying the source of bleeding. Computerized tomography angiography (CTA) revealed a mass lesion in the jejunum with active intravenous contrast extravasation (Figure 1). The patient’s unstable condition determined immediate intervention. In order to accurately mark the location of the lesion, thereby avoiding an exploratory laparotomy, the patient underwent diagnostic angiography of the superior mesenteric artery arcade system, revealing a hypervascular jejunal mass (Figure 2). Selective metal coil embolization of the feeding vessel was performed. On the same day the patient was taken to the operating theatre for a laparoscopic exploration of the abdomen. A 2.5 cm tumor arising from the anti-mesenteric border of the proximal jejunum was identified (Figure 3). The embolization coil was visualized adjacent to the bowel wall, with segmental ischemic discoloration of the serosa. Small bowel resection with primary anastomosis was performed. Postoperative course was uneventful. Pathology results showed a CD 117 positive gastrointestinal stromal tumor with a low MIB-1 proliferation index of 1% (Figure 4). Surgical margins were free of tumor. Small erosions were noted in the adjacent mucosa, the most probable source of bleeding.

The patient was discharged in good overall medical condition. Medical therapy (tyrosine kinase inhibitors) was not started due to the low chance of GIST recurrence.

## Conclusions

GIST is the most common sarcoma of the GI tract with an incidence of 10–20 per million, accounting for 0.3- 0.5% of all GI tumors [1, 4]. GISTs are most commonly found in the stomach (60%), followed by the small intestine (30%), large bowel (5%), and rectum (5%) [1, 2, 5].

These tumors were once considered to be leiomyomas, leiomyosarcomas, leiomyoblastomas or schwanommas, based on their histology; however, it has become evident that these tumors are a distinct entity caused by specific gain of function mutations in the C-Kit, tyrosine kinase protein [6]. It has been proposed that the cellular origin of GISTs is the interstitial cell of Cajal, the pacemaker of the alimentary tract [5]. Cajal cells exhibit both neuronal and smooth muscle cell histological features that are frequently found in GIST.

The majority of patients with GIST present with vague symptoms such as abdominal pain, GI bleeding or abdominal fullness [2].

Obscure GI bleeding (OGIB) is defined as bleeding which persists despite negative findings in bi-directional endoscopy [7]. OGIB can be divided into two types: *overt*, when bleeding is clinically evident as in our case; and *occult*, when the manifestation of bleeding is anemia or positive fecal occult blood. Pathological processes along the entire GI tract can cause OGIB.

Video capsule endoscopy (VCE) is considered the preferred diagnostic tool in these patients because of its high sensitivity [7, 8]; due to the time it takes to perform the test and analyze the video footage, however, VCE is often not a practical option in patients with active bleeding, as in the case presented here. Additionally, double balloon assisted enteroscopy (DBE) and spiral enteroscopy can be performed. The diagnostic yield of DBE for small bowel mesenchymal tumors has been reported to be nearly 90% [9], but this case series included only one patient with overt bleeding. A labeled red blood cell nuclear scan can detect a source of bleeding that has a low rate of blood loss (less than 0.5 ml/min), but poorly demonstrates the anatomic location of the bleeding source. A labeled pertechnetate or Meckel’s scan is useful in diagnosing Meckel’s diverticulum as the bleeding source, but may show false negative results during active bleeding [7]. Angiography is useful in detecting active bleeding with rates above 0.5 ml/min, as well as vascular lesions not actively bleeding. The main advantage of angiography over other modalities is the ability to treat as well as diagnose OGIB. Due to the invasiveness and potential complications, exploratory surgery is a last resort for detecting the cause and location of OGIB. Any of the aforementioned modalities can be combined in the work up and diagnosis of OGIB. Current treatment recommendations include surgical excision with or without imatinib mesylate therapy, depending on the likelihood of recurrence.

Our patient presented with symptomatic GI bleeding. Endoscopy was normal and colonoscopy demonstrated passage of blood without finding a bleeding source. The combination of these findings led to suspicion of a small intestinal bleeding source. Computerized tomography (CT) enterography or angiography is a useful tool that can accurately localize a bleeding source. Some authors argue that CT should be part of the routine diagnostic work up of small bowel occult GI bleeding, and may be more sensitive than VCE in select cases [8]. Additionally, CT is often more readily available than capsule endoscopy in certain clinical settings. Both tests are considered acceptable third-line investigations in the diagnosis of obscure GI bleeding following bi-directional endoscopy [7]. Angiography was used both to treat acute bleeding, by placement of a coil in the feeding vessel, and to mark the location of the bleeding lesion for upcoming surgery. During surgery an exophytic tumor of the jejunum was seen with segmental ischemic discoloration of the serosa.

We believe that this case report of an uncommon etiology of obscure GI bleeding demonstrates both the importance of CT in the diagnosis of small bowel obscure bleeding and the efficacy of angiography in addition to surgery in the treatment of small bowel obscure bleeding.

Massive overt GI bleeding is an atypical presentation of small bowel GIST. CT is a useful tool for initial diagnosis and quick localization of submucosal alimentary tumors in patients with active bleeding. Selective embolization is an important diagnostic and therapeutic modality in actively bleeding small bowel GIST, but carries the risk of bowel necrosis. Complete surgical resection remains the mainstay of treatment of GIST.

## Consent

Written informed consent was obtained from the patient for publication of this case report and any accompanying images. A copy of the written consent is available for review by the editor of this journal.

## Abbreviations

CT: Computerized tomography
CTA: Computerized tomography angiography
ED: Emergency department
EGD: Esophago-gastro-duodenoscopy
GI: Gastrointestinal
GIST: Gastrointestinal stromal tumor
OGIB: Occult gastrointestinal bleeding
SMA: Superior mesenteric artery
VCE: Video capsule endoscopy.
